# Intrinsically Microporous Polyimides Derived from 2,2′-Dibromo-4,4′,5,5′-bipohenyltetracarboxylic Dianhydride for Gas Separation Membranes

**DOI:** 10.3390/polym16091198

**Published:** 2024-04-25

**Authors:** Yongle Li, Yao Lu, Chun Tian, Zhen Wang, Jingling Yan

**Affiliations:** 1 Ningbo Institute of Material Technology & Engineering, Chinese Academy of Sciences, Ningbo 315201, China; liyongle@nimte.ac.cn (Y.L.); wz@nimte.ac.cn (Z.W.); jyan@nimte.ac.cn (J.Y.); 2 College of Chemical Engineering, Zhejiang University of Technology, Hangzhou 310014, China; 3 School of Fine Arts, Zhengzhou University, Zhengzhou 450001, China; luyao94@zzu.edu.cn

**Keywords:** PIM-PIs, 2,2′-dibromo-4,4′,5,5′-bipohenyltetracarboxylic dianhydride, interchain distances, bromo group, gas separation performance

## Abstract

This work aims to expand the structure–property relationships of bromo-containing polyimides and the influence of bromine atoms on the gas separation properties of such materials. A series of intrinsically microporous polyimides were synthesized from 2,2′-dibromo-4,4′,5,5′-bipohenyltetracarboxylic dianhydride (Br-BPDA) and five bulky diamines, (7,7′-(mesitylmethylene)bis(8-methyldibenzo[b,e][1,4]dioxin-2-amine) (MMBMA), 7,7′-(Mesitylmethylene)bis(1,8-dimethyldibenzo[b,e][1,4] dioxin-2-amine) (MMBDA), 4,10-dimethyl-6H,12H-5,11-methanodibenzo[b,f][1,5]diazocine-2,8-diamine (TBDA1), 4,10-dimethyl-6H,12H-5,11-methanodibenzo[b,f][1,5]diazocine-3,9-diamine (TBDA2), and (9R,10R)-9,10-dihydro-9,10-[1,2]benzenoanthracene-2,6-diamine (DAT). The Br-BPDA-derived polyimides exhibited excellent solubility, high thermal stability, and good mechanical properties, with their tensile strength and modulus being 59.2–109.3 MPa and 1.8–2.2 GPa, respectively. The fractional free volumes (*FFVs*) and surface areas (*S*_BET_) of the Br-BPDA-derived polyimides were in the range of 0.169–0.216 and 211–342 m^2^ g^−1^, following the order of MMBDA > MMBMA > TBDA2 > DAT > TBDA1, wherein the Br-BPDA-MMBDA exhibited the highest *S*_BET_ and *FFV* and thus highest CO_2_ permeability of 724.5 Barrer. Moreover, Br-BPDA-DAT displayed the best gas separation performance, with CO_2_, H_2_, O_2_, N_2_, and CH_4_ permeabilities of 349.8, 384.4, 69.8, 16.3, and 19.7 Barrer, and H_2_/N_2_ selectivity of 21.4. This can be ascribed to the ultra-micropores (<0.7 nm) caused by the high rigidity of Br-BPDA-DAT. In addition, all the bromo-containing polymers of intrinsic microporosity membranes exhibited excellent resistance to physical ageing.

## 1. Introduction

Polyimides (PIs) have been considered as one of the most attractive and promising gas separation membrane materials due to their good film-forming properties, excellent heat and chemical resistance, good mechanical properties, and high gas perm-selectivity. Commercial polyimides for gas separation membranes include Matrimid^®^5218, P84^®^, Upilex^®^, etc. [1]. However, the traditional PIs exhibit dense chain packing, resulting in a low fractional free volume (*FFV*) and insufficient gas permeability. Thus, their gas separation performances were below the Robeson’s upper bounds [2,3,4].

So far, there have been several emerging polymers that possess enhanced gas permeability, e.g., the polymers of intrinsic microporosity (PIMs) [5,6,7]. The excellent gas separation performance of PIMs can be explained by their twisted and highly inflexible polymer backbones, which leads to the formation of unique micropores of <2 nm and ultra-micropores of <0.7 nm [8,9]. Various PIMs have been exploited to increase polymer rigidity by incorporating bulky and stiff moieties [10], such as spirobisindane (SBI) [11], Tröger’s base (TB) [12], and triptycene (Trip) [13]. The introduction of micropores into PIs enhanced gas permeability, although a decrease in selectivity was often observed. Many PIMs have overcome the trade-off relationship and surpassed the Robeson’s upper bounds [4,14,15]. Nonetheless, PIMs still suffer several disadvantages, such as a complicated synthetic procedure, an extremely high cost, inferior mechanical properties, and poor resistance to physical ageing and CO_2_-induced plasticization. These limitations hamper their widespread application in gas separation [11,16,17,18]. In addition, nanoparticles were also introduced into PIMs, which can also improve the CO_2_ diffusion coefficients of the membranes, thereby enhancing the separation performance [19,20,21].

At present, 3,3′,4,4′-biphenyltetracarboxylic dianhydride (4,4′-BPDA) is an extensively used commercial monomer for high-performance PIs. 4,4′-BPDA-derived PIs often exhibit strong intramolecular or intermolecular interactions, such as π-π stacking, which cause high gas selectivity [22]. Tanaka et al. investigated the gas separation performance of polyimides prepared from 4,4′-BPDA and 4,4′-oxydianiline (ODA). The results showed that 4,4′-BPDA-ODA displayed a H_2_/CH_4_ and CO_2_/CH_4_ selectivity of 173 and 29, respectively [23]. However, 4,4′-BPDA-derived PIs showed relatively low gas permeability, which is attributed to strong intermolecular interactions leading to low *d*-spacing of the resulting polymers. This limitation can be addressed by the incorporation of bulky 2,2′-positioned substituents in 4,4′-BPDA, which can increase the *d*-spacing and *FFV* of the resulting polymers. Li et al. reported a series of PIs derived from 2,2′-disubstituted BPDAs [24,25]. The introduction of bulky substituents at the 2,2′ positions of 4,4′-BPDA restricted the rotation of the single bonds between two phthalimide segments and thus increased chain rigidity. Furthermore, the bulky substituents in the main chain generally tend to increase *FFV* and hence gas permeability [26]. Kwon et al. reported a series of PIs from commercial diamines, 2,2′-bis(4′′-tert-butylphenyl)-4,4′,5,5′-biphenyltetracarboxylicdianhydride and 2,2′-bis(4′′-trimethylsilylphenyl)-4,4′,5,5′-biphenyltetracarboxylic dianhydride [27]. The gas permeability in these PIs was significantly enhanced by the introduction of bulky 4′′-tert-butylphenyl or 4′′-trimethylsilylphenyl substituents, with their O_2_ permeability and O_2_/N_2_ selectivity being 31–110 Barrer and 2.8–4.3, respectively. Zhang et al. reported PIs from 2,2′-phenoxyl-substituted BPDA [26]. The results indicated that O_2_ permeability increased with the size of the substituents, following the order of *tert*-butyl phenoxyl > methyl phenoxyl > phenoxyl, while their O_2_/N_2_ selectivity followed the opposite trend, ranging from 3.8 to 4.6. Bromine has a larger Van der Waals volume (V_w_) of 14.60 cm^3^ mol^−1^ than many other groups, such as -Cl (12.00 cm^3^ mol^−1^), -CH_3_ (13.67 cm^3^ mol^−1^), -NH_2_ (7.44 cm^3^ mol^−1^) and -OH (8.00 cm^3^ mol^−1^). Recently, Zhao and co-workers synthesized PIM-PIs derived from dibromo substituted fluorine-containing diamines [28]. The results showed that introducing bromine substituents can considerably improve the ability to resist physical aging. Furthermore, the high V_w_ value of the bromo group is conducive to improving gas permeability [29].

Hence, in this work, we reported PIM-PIs from 2,2′-dibromo- 4,4′,5,5′-bipohenyltetracarboxylic dianhydride (Br-BPDA) and five typical twisted diamines, i.e., (7,7′-(mesitylmethylene)bis(8-methyldibenzo[b,e][1,4]dioxin-2-amine) (MMBMA), 7,7′-(Mesitylmethylene)bis(1,8-dimethyldibenzo[b,e][1,4] dioxin-2-amine) (MMBDA), 4,10-dimethyl-6H,12H-5,11-methanodibenzo[b,f][1,5]diazocine-2,8-diamine (TBDA1), 4,10-dimethyl-6H,12H-5,11-methanodibenzo[b,f][1,5]diazocine-3,9-diamine (TBDA2), and (9R,10R)-9,10-dihydro-9,10-[1,2]benzenoanthracene-2,6-diamine (DAT). A systematic study was conducted on the physical properties, microporous characteristics, and *FFV* of these polymers, and their performances regarding gas separation were evaluated.

## 2. Materials and Methods

### 2.1. Materials

4,4′-BPDA was obtained from China Tech Chemical Co., Ltd. (Tianjin, China), and dried at 250 °C under vacuum prior to use. *N*-Methylpyrrolidone (NMP), *m*-cresol, and ethanol were purchased from Energy Chemical Co., Ltd. (Shanghai, China). *m*-cresol and NMP were distilled over calcium hydride and stored in argon-purged bottles. All other chemicals were obtained from J&K Scientific Ltd. (Beijing, China) and were used as received. The synthesis methods of Br-BPDA [30], MMBMA, MMBDA [31], TBDA1, TBDA2 [12], and DAT [32] came from publications.

### 2.2. Polymer Synthesis

The Br-BPDA-derived polyimides were synthetized by a one-step solution polycondensation method in *m*-cresol.

Br-BPDA-DAT: Br-BPDA (0.9002 g, 2.00 mmol), DAT (0.5688 g, 2.00 mmol), benzoic acid (0.5446 g, 4.00 mmol), and *m*-cresol (3.4 mL) were added in a 20 mL flask with three-necked under nitrogen at 90 °C. Once the mixture became homogeneous, the reaction temperature was raised to 190 °C and held for 6 h. After cooling to 100 °C, the resulting viscous solutions were poured slowly into a mixture of methanol (50.0 mL) and deionized water (50.0 mL). The fibrous polymers were collected and purified by re-dissolution in chloroform and precipitation into methanol thrice. In a Soxhlet extractor, the PIs were then purified with methanol for 48 h and dried under vacuum at 140 °C for 8 h to obtain Br-BPDA-DAT (0.6978 g, yield: 95%). ^1^H NMR (400 MHz, DMSO-*d*_6_): δ 8.40 (s, 2H), 7.94 (s, 2H), 7.63–7.52 (m, 6H), 7.14–7.05 (m, 4H), 5.84 (s, 2H). FT-IR: 2969, 2917, 2858 cm^−1^ (C-H), 1781 cm^−1^ (C=O), 1728 cm^−1^ (C=O), 1376 cm^−1^ (C-N-C), 598 cm^−1^ (C-Br).

Br-BPDA-MMBDA: This polymer was prepared according to a procedure similar to Br-BPDA-DAT, yield: 88%. ^1^H NMR (400 MHz, CDCl_3_): δ 8.27 (s, 2H), 7.78 (s, 2H), 6.82–6.74 (m, 8H), 6.35 (s, 2H), 5.46 (s, 1H), 2.26–1.68 (m, 21H). FT-IR: 2969, 2917, 2858 cm^−1^ (C-H), 1781 cm^−1^ (C=O), 1728 cm^−1^ (C=O), 1376 cm^−1^ (C-N-C), 598 cm^−1^ (C-Br).

Br-BPDA-MMBMA: This polymer was prepared according to a procedure similar to Br-BPDA-DAT, yield: 93%. ^1^H NMR (400 MHz, CDCl_3_): δ 8.30 (s, 2H), 7.80 (s, 2H), 6.95–6.68 (m, 10H), 6.34 (s, 2H), 5.45 (s, 1H), 2.25–1.98 (m, 15H). FT-IR: 2969, 2917, 2858 cm^−1^ (C-H), 1780 cm^−1^ (C=O), 1728 cm^−1^ (C=O), 1376 cm^−1^ (C-N-C), 598 cm^−1^ (C-Br).

Br-BPDA-TBDA1: This polymer was prepared according to a procedure similar to Br-BPDA-DAT, yield: 89%. ^1^H NMR (400 MHz, CDCl_3_): δ 8.26 (s, 2H), 7.78 (s, 2H), 7.11 (s, 2H), 6.86 (s, 2H), 4.66 (d, 2H), 4.33 (s, 2H), 4.08 (d, 2H), 2.45 (s, 6H). FT-IR: 2969, 2917, 2858 cm^−1^ (C-H), 1781 cm^−1^ (C=O), 1728 cm^−1^ (C=O), 1376 cm^−1^ (C-N-C), 599 cm^−1^ (C-Br).

Br-BPDA-TBDA2: This polymer was prepared according to a procedure similar to Br-BPDA-DAT, yield: 92%. ^1^H NMR (400 MHz, CDCl_3_): δ 8.35 (d, 2H), 7.87 (d, 2H), 7.00 (s, 4H), 4.70 (d, 2H), 4.38 (s, 2H), 4.12 (d, 2H), 2.30 (d, 6H). FT-IR: 2969, 2917, 2858 cm^−1^ (C-H), 1780 cm^−1^ (C=O), 1728 cm^−1^ (C=O), 1376 cm^−1^ (C-N-C), 599 cm^−1^ (C-Br).

### 2.3. Membrane Casting

The chloroform solutions (3 wt%) of the Br-BPDA-derived polymers were purified through 1 μm PTFE filters and cast onto glass panels. They were dried at room temperature for three days to remove chloroform. Specially, Br-BPDA-DAT was dissolved in NMP (3 wt%), and the solvent was depleted by drying at 60 °C for 5 h, 150 °C for 1 h, 200 °C for 1 h, and 250 °C for 4 h in vacuum.

### 2.4. Characterization

Fourier-transform infrared (FT-IR) spectra and ^1^H NMR spectra were obtained on a Cary660+620 Micro FTIR instrument (Agilent, Santa Clara, CA, USA) and Bruker Advance Neo 600 spectrometer (Bruker, Rheinstetten, Germany), respectively. Number average molecular weights (*M*_n_), weight average molecular weights (*M*_w_), and polydispersity indices (PDI) were measured on TOSOH HLC-8420GPC (TOSOH, Tokyo, Japan) gel permeation chromatography (GPC) equipped with a refractive index detector using dimethylformamide (DMF + LiBr 0.1 wt%) as the eluent at a flow rate of 0.3 mL min^−1^ and polystyrene as the calibration standard at 40 °C. Wide-angle X-ray diffraction (WAXD) was performed on a Bruker D8 Advance Davinci instrument (Bruker, Karlsruhe, Germany) with Cu Kα radiation (λ = 1.54 Å) and an angular range from 5° to 50°. The storage modulus and tan δ of the Br-BPDA-derived PIM-PIs were studied using a Q850 DMA (TA Instruments, New Castle, DE, USA) at a heating rate of 5 °C min^−1^. Thermogravimetric analysis (TGA) was performed on a Q55 TGA (TA Instruments, New Castle, DE, USA) at a heating rate of 10 °C min^−1^ in nitrogen. Brunauer–Emmett–Teller surface areas (*S*_BET_) were measured via N_2_ adsorption at 77 K using an ASAP 2460 instrument (Micromeritics Instrument Corporation, Norcross, GA, USA). Tensile testing was performed on a TCS-2000 electron tensile testing machine (Gotech Testing Machines Inc., Taichung City, Taiwan) at a constant displacement rate of 2 mm min^−1^. Contact angles were obtained using a sessile drop water with a Dataphysics OCA-20 contact angle analyzer. The density of the Br-BPDA-derived PIM-PIs were obtained using an SQP balance (Sartorius, Gottingen, Germany) equipped with a density measurement kit. The *FFV* of the Br-BPDA-derived PIM-PIs was calculated using the group contribution method in the literature [10,33]. The gas permeabilities of the Br-BPDA-derived PIM-PIs were measured on a PERME VAC-2 permeation system (Labthink Instruments Co., Ltd., Jinan, China) at 1 bar and 35 °C [34].

## 3. Results and Discussion

### 3.1. Synthesis of Polymers

**Scheme 1:**
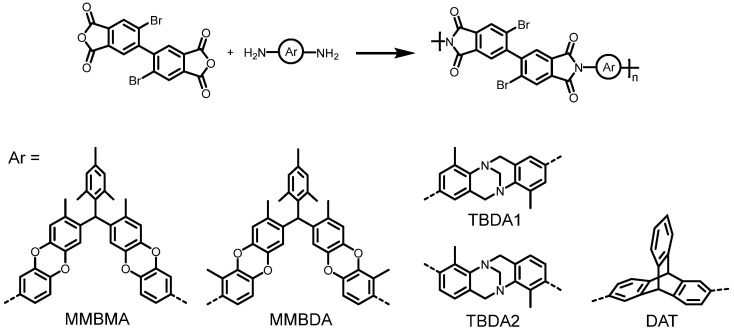
Synthesis of Br-BPDA-derived polymers.

According to previous reports, polyimides derived from 2,2′-disubstituted BPDA exhibit better solubility than those based on 4,4′-BPDA [24,25]. And, given the close V_w_ values of Br-BPDA and 6FDA (161.12 vs. 184.75 cm^3^ mol^−1^) [35], it could be anticipated that that polyimides from Br-BPDA have a similar *FFV* to those based on 6FDA. Meanwhile, MMBMA, MMBDA, TBDA1, TBDA2, and DAT have been extensively used for the preparation of PIM-PIs with a high *FFV* [12,31,32]. In this work, PIM-PIs were obtained by combining Br-BPDA with five typical rigid and twisted diamines through high-temperature solution polymerization (Scheme 1). The *M*_w_ and PDI values of these polymers were 18.0–45.0 kg mol^−1^ and 1.8–3.8 (Table 1), respectively. Br-BPDA-MMBMA exhibited the highest molecular weight, which may be attributed to the high reactivity of MMBMA. The FT-IR spectra of the Br-BPDA-derived polymers are shown in Figure S1, and the successful synthesis of the imine ring structure are demonstrated by the characteristic peaks at ~1770 cm^−1^ (asymmetric C=O stretching), ~1730 cm^−1^ (symmetric C=O stretching), and ~1360 cm^−1^ (C-N stretching). Moreover, the absorption peaks of amide or amino groups, located at ~3350 cm^−1^ (asymmetric N-H stretching vibration), ~3170 cm^−1^ (symmetric N-H stretching), ~1650 cm^−1^ (N-H bending) completely disappear, indicating the complete reaction of diamine with no residual diamine molecules in the polymer chain. And, in the ^1^H NMR spectra (Figure S2), the peaks at 8.3 ppm and 7.8 ppm represent the aromatic ring protons in the Br-BPDA residues. The absence of peaks for -NH_2_ and -COOH groups also proves the successful preparation of polyimides. Additionally, the chemical structure of Br-BPDA-DAT is further confirmed by ^13^C NMR (Figure S4), where the features are fully assigned. These results confirm the successful synthesis of Br-BPDA-derived PIM-PIs. 

**Figure 1:**
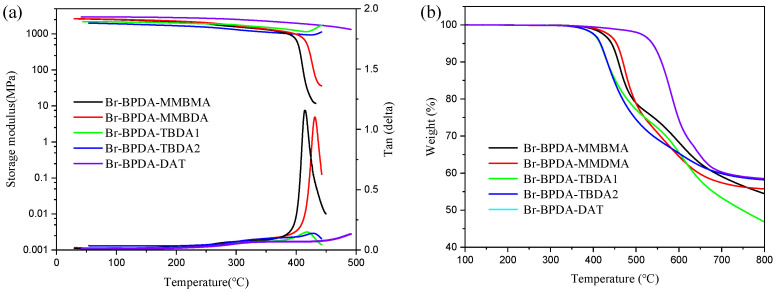
(**a**) DMA curves and (**b**) TGA curves of Br-BPDA-derived PIM-PIs.

All the Br-BPDA-derived PIs exhibited good solubility in high-boiling-point solvents due to the introduction of bromine substituents (Table S1). In addition, the PIs with TB and dibenzodioxane segments were soluble in low-boiling-point solvents like CHCl_3_. In contrast, Br-BPDA-DAT exhibited relatively poor solubility in the overall series perhaps due to the unique Trip structure in DAT leading to a higher aromatic ring content. Due to the lower chain packing density (Table 2), Br-BPDA-MMBDA and Br-BPDA-MMBMA showed the best solubility. These polymers showed high modulus (59.2–109.3 MPa) and good strength (1.8–2.2 GPa), indicating that they have outstanding mechanical properties (Table 1 and Figure S4). The glass transition temperatures (*T*_g_) of these polymers were obtained by DMA testing (Figure 1), and all *T*_g_ values were higher than 400 °C. In particular, the *T*_g_ of Br-BPDA-DAT was higher than 500 °C. The *T*_g_ values of these PIM-PIs followed the order of Br-BPDA-MMBMA < Br-BPDA-TBDA1 < Br-BPDA-TBDA2 < Br-BPDA-MMBDA < Br-BPDA-DAT. Br-BPDA-MMBMA exhibited the lowest *T*_g_ due to its relatively low chain rigidity, resulting from the absence of *ortho*-positioned methyl substituents.

The contact angles of the Br-BPDA-derived PIM-PIs were 95.0–103.9° (Figure S5). The hydrophobicity of these polymers can be explained by their relatively lower imide contents, as well as the methyl substituents for some cases.

### 3.2. Microstructural Properties

**Figure 2:**
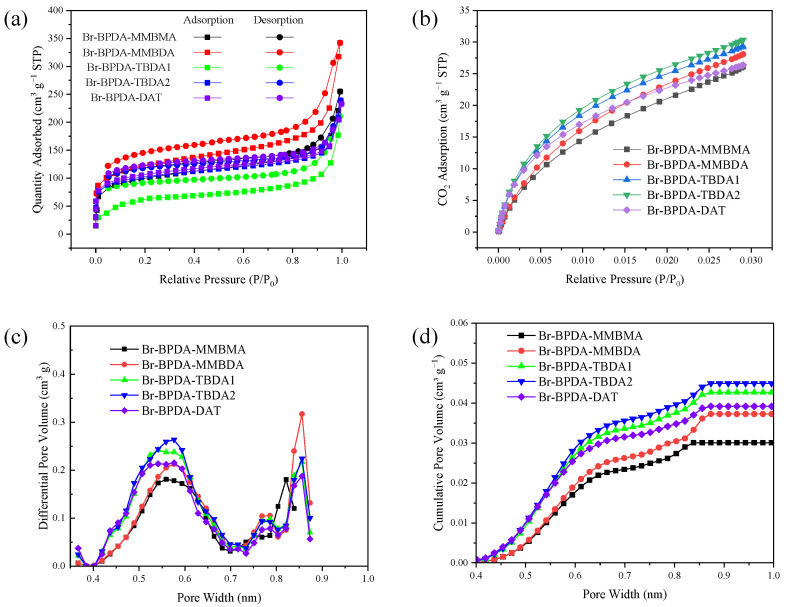
(**a**) N_2_ adsorption/desorption isotherms, (**b**) CO_2_ absorption, (**c**) differential pore volume, (**d**) cumulative pore volumes of Br-BPDA-derived PIM-PIs.

It can be observed that there were significant hysteresis loops and a higher nitrogen absorption at a relatively low pressure in the nitrogen adsorption desorption isotherm, both of which were obvious characteristics of intrinsic microporous polymers (Figure 2a) [11]. The values of *S*_BET_ ranged from 211 to 342 m^2^ g^−1^, with the order of Br-BPDA-TBDA1 < Br-BPDA-DAT < Br-BPDA-TBDA2 < Br-BPDA-MMBMA < Br-BPDA-MMBDA. And, the value of *S*_BET_ for Br-BPDA-MMBDA was 21% higher than that of Br-BPDA-MMBMA, and the value of *S*_BET_ for Br-BPDA-TBDA2 was 64% higher than that of Br-BPDA-TBDA1, which is also consistent with the value of the *FFV*. It can be deduced that the introduction of bulky bromine atoms and *ortho*-positioned methyl group will restrain the chain densification of PI, open the polymer backbone, and induce a larger free volume [23].

The CO_2_ uptake is affected by the CO_2_ affinity and specific surface areas of polymers [2]. For these Br-BPDA-derived PIM-PIs, at 273 K and P/P_0_ = 0.029, the CO_2_ uptake ranged from 26.1 to 30.4 cm^3^ g^−1^ (Figure 2b and Table 2). Among them, Br-BPDA-TBDA1/TBDA2 exhibited a higher CO_2_ uptake because of the dipole–quadrupole interaction between the tertiary amine in the TB skeleton and the polarized CO_2_ molecule [12,36,37,38]. This result also demonstrates that *ortho*-positioned methyl groups have a similar effect on the surface areas of polymers, like Br-BPDA-TBDA1(29.3 cm^3^ g^−1^) < Br-BPDA-TBDA2 (30.4 cm^3^ g^−1^) and Br-BPDA-MMBMA (26.1 cm^3^ g^−1^) < Br-BPDA-MMBDA (28.1 cm^3^ g^−1^). This indicates that *ortho*-substituted groups (-Br and -CH_3_) hinder rotational chemical bonds and have an impact on the surface, which is consistent with the *FFV* and *S*_BET_ results.

The cumulative volume of micropores (V_M_) for these polymers spanned a range of 0.030–0.045 cm^3^ g^−1^. Br-BPDA-MMBDA/MMBMA showed a relatively lower V_M_ value, owing to the relatively low rigidity of dibenzodioxane moieties relative to TB or Trip segments. In addition, the V_M_ values of Br-BPDA-TBDA1 were lower than Br-BPDA-TBDA2, and the V_M_ values of Br-BPDA-MMBMA were lower than Br-BPDA-MMBDA, respectively, due to the absence of an *ortho* substituents methyl group.

**Figure 3:**
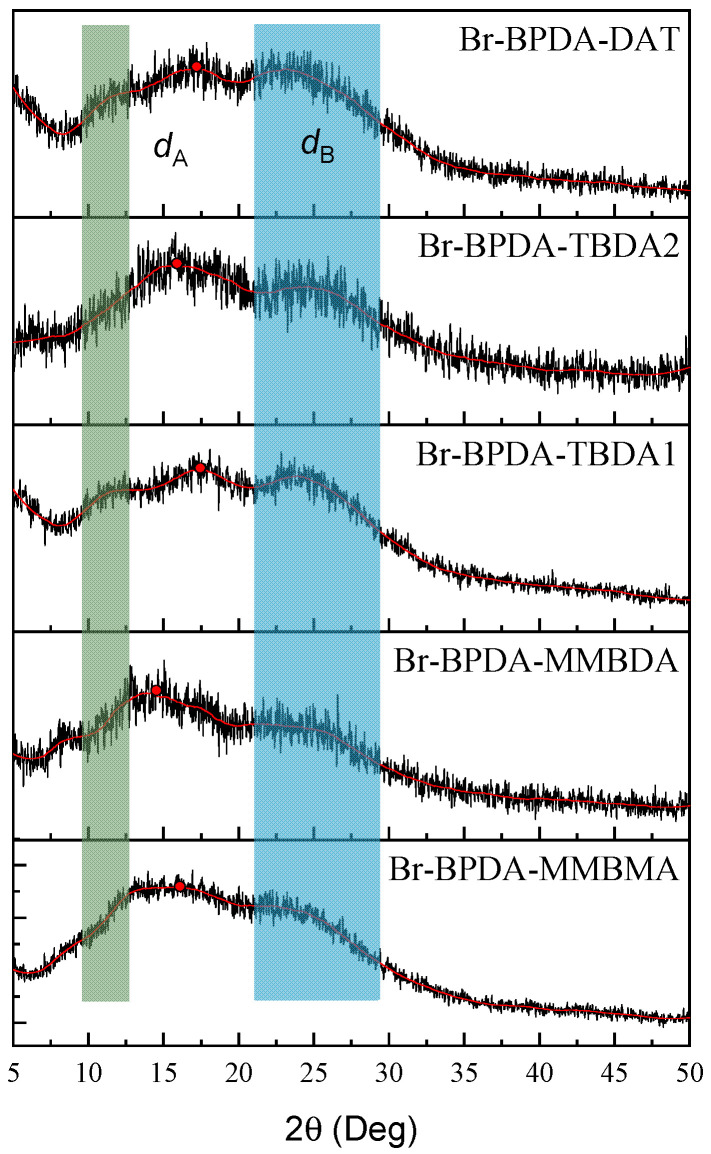
WAXD patterns of Br-BPDA-derived PIM-PIs (Black line: Experimental data; Red line: Linear fitting curve of experimental data; Red point: The highest peak at *d*_A_).

Regarding density, according to Bondi’s group contribution method [35,39], the values of the *FFV* of these polyimides, ranged from 0.169 to 0.216 (Table 2). And, similar tendencies were observed for the *FFV* and *S*_BET_ of these polymers. The chain packing profiles of the Br-BPDA-derived PIM-PIs were characterized by WAXD measurements (Figure 3 and Table 2). The diffraction peaks were mainly located in the range of 10–30°, indicating their amorphous features. The average distances between different molecular chains of the corresponding PIM-PIs were in the range of 4.92–5.88 and 3.75–3.94 Å, respectively. Two major peaks were fitted in these diffraction peaks (labelled as *d*_A_ and *d*_B_) [40,41], where *d*_A_ and *d*_B_ were respectively assigned to the interchain distances and the π-π stacking interactions. The effect of *ortho* substituents on d-spacing was also observed in WAXD curves, the *d*_A_ value of Br-BPDA-MMBMA < Br-BPDA-MMBDA, and the *d*_A_ value of Br-BPDA-TBDA1 < Br-BPDA-TBDA2. Additionally, a shoulder peak was also observed at around 10°, which corresponded to the larger micropores in the PIM-PIs. The result is beneficial for improving gas permeability. The amorphous feature was a typical characteristic of the PIM-PI that could be applied to gas separation. This tendency was consistent with the *FFV* and *S*_BET_ of the polymers.

### 3.3. Gas Separation Performance

The water and CO_2_ in the air can cause the film to plasticize, increasing the *FFV* and reducing the gas selectivity of the polymers [42]. So, before conducting gas separation tests, it was necessary to soak the fresh membranes in methanol for one day, which was beneficial for improving the gas separation performance [43], and then dry them under vacuum at 100 °C to remove methanol and H_2_O from the membranes. The pure gas transport properties of these PIs were investigated with gases including H_2_, CO_2_, O_2_, N_2_, and CH_4_. Their gas permeabilities and selectivities are shown in Table 3. For the given polymers, the gas permeabilities of these polymers were in the order of P_CO_2__ > P_H_2__ > P_O_2__ > P_CH_4__ > P_N_2__. However, after ageing for 300 or 900 days, the order between P_CH_4__ and P_N_2__ was reversed. For instance, the P_H_2__, P_CO_2__, P_O2_, P_N2_, and P_CH_4__ of Br-BPDA-MMBDA (*FFV* = 0.216) were 576.5, 724.5, 143.2, 42.9, and 61.7 Barrer, respectively, which were 1.6–3.1 times greater than those of Br-BPDA-DAT (*FFV* = 0.189). In addition, Br-BPDA-TBDA1 (*FFV* = 0.169), Br-BPDA-TBDA2 (*FFV* = 0.177), and Br-BPDA-DAT (*FFV* = 0.189) showed moderate gas permeabilities despite their smaller *FFV* because of the Trip and TB groups, leading to higher rigidity. However, compared with Br-BPDA-TBDA1, Br-BPDA-TBDA2 was more permeable due to the presence of *ortho*-substituted methyl groups, leading to insufficient space for the free rotation of C-N bonds in the imide rings. Br-BPDA-MMBDA exhibited the lowest selectivity due to its moderate *FFV* but a low cumulative micropore volume (Figure 2c). In contrary, Br-BPDA-TBDA1 displayed the highest selectivity because of the lowest d-spacing (*d*_A_ = 4.92 Å) and *FFV*. In these PIs containing TB or Trip structures, the unique rigid structure gave the polymer a higher cumulative volume of ultramicropores, while strong π-π stacking interactions gave the polymer a higher gas sieving ability [44]. The synergistic effect of these two factors maintained a good balance between permeability and selectivity.

**Figure 4:**
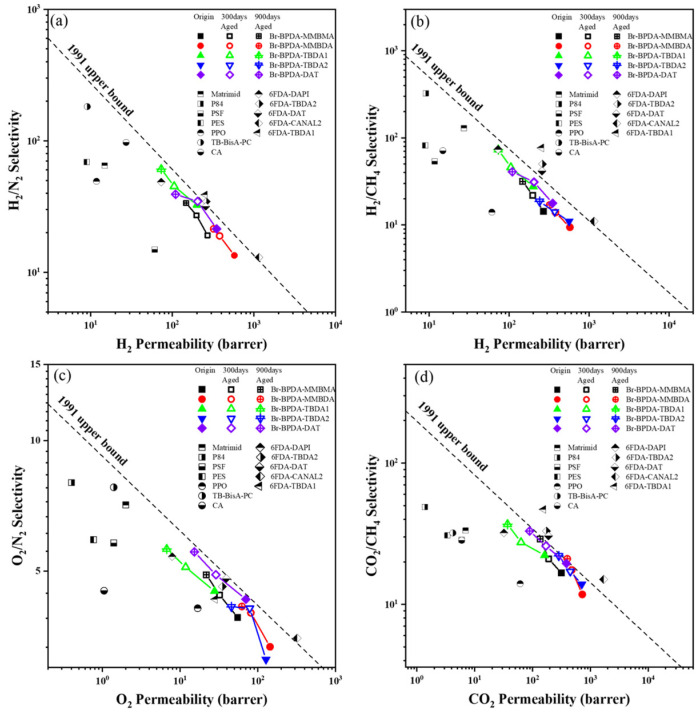
Robeson plots of (**a**) H_2_/N_2_, (**b**) H_2_/CH_4_, (**c**) O_2_/N_4_, and (**d**) CO_2_/CH_4_ for Br-BPDA-derived PIM-PIs.

The gas separation performance of the Br-BPDA-derived PIM-PIs with other commercial membranes was also compared according to Robeson’s upper bounds (Figure 4). The Br-BPDA-derived PIM-PIs exhibited better performances than most commercial polymers, such as Matrimid^®^ 5218, P84^®^ [3,44,45,46,47,48,49,50,51,52,53]. However, the Br-BPDA-derived polymers showed a slightly inferior or similar performance regarding gas separation compared to their counterparts based on 6FDA due to the slightly lower V_w_ of Br-BPDA than 6FDA (161.12 vs. 184.75 cm^3^ mol^−1^) [12,39,42,45].

**Figure 5:**
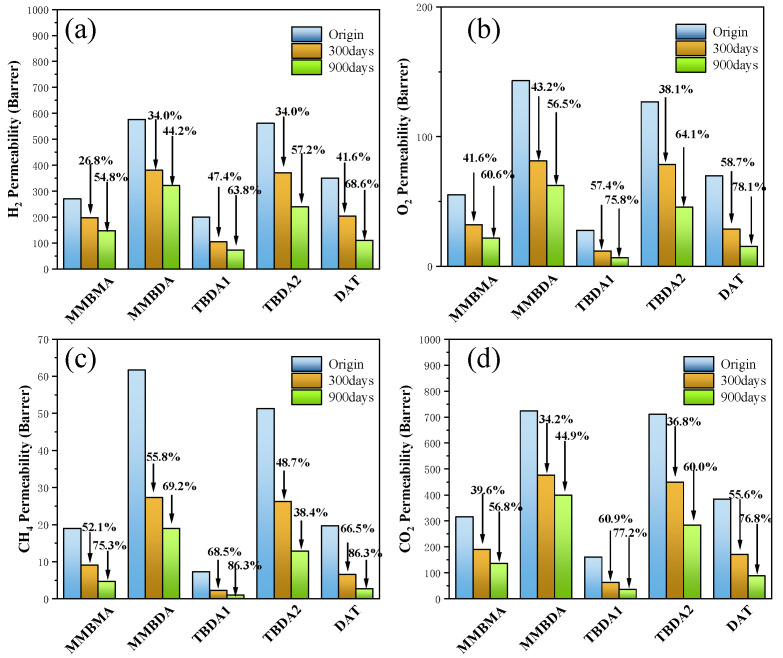
Effect of physical aging time on gas permeability of Br-BPDA-derived PIM-PIs, (**a**) H_2_, (**b**) O_2_, (**c**) CH_4_, (**d**) CO_2_.

The physical aging characteristics of the Br-BPDA-derived PIM-PIs were systematically assessed. The gas separation performances of fresh membranes and those aged membranes were shown in Figure 4 and Table 3. The permeabilities of all the PIM-PIs decreased, and the selectivities increased due to the collapse of larger micropores and the decrease in *FFV*. The loss in gas permeability was most pronounced in gases with larger kinetic diameters, resulting in the improved selectivity of H_2_/N_2_, H_2_/CH_4_, and CO_2_/CH_4_. Zhao et al. [28,29] investigated the effect of -Br groups on the physical aging of polymer membranes and found that the bromo groups could effectively retard the speed of physical aging by the interference with the nearby carbonyl groups and restricted rotation. Similar trends were also observed in this work. The H_2_ permeability of all the Br-BPDA-derived PIM-PIs decreased by only 26.7–41.5% after aging for 300 days and 45.2–68.6% for 900 days (Figure 5). In addition, the gas separation performance of all the aged membranes was well retained or even improved compared to the fresh membranes, indicating their excellent resistance to physical ageing.

## 4. Conclusions

Five high-molecular weight PIM-PIs were prepared from Br-BPDA and different diamines (MMBMA, MMBDA, TBDA1, TBDA2, and DAT) via the “one-step” method in *m*-cresol. These Br-BPDA-derived PIM-PIs exhibited extremely high *T*_g_ (>400 °C) because of their twisted structure from diamines and *ortho*-substituted groups (-Br and -CH_3_) hinder rotational chemical bonds. And, the Br-BPDA-derived PIM-PIs showed favorable mechanical properties for gas separation application, tensile strength > 59.2 MPa, modulus > 1.8 GPa, and elongation at break > 3.4%. The markedly high *d*-spacing values of the polymers (>0.492 nm) were observed due to the incorporation of large bromine substituents and a twisted diamine monomer. Br-BPDA-MMBDA displayed the highest gas permeability but lowest perm-selectivity due to its highest *FFV* (0.216) and lowest cumulative volume of micropores, leading to its relatively poor gas separation performance. On the contrary, Br-BPDA-DAT showed high perm-selectivity while maintaining moderate gas permeability due to the high rigidity and unique internal *FFV* of Trip moieties. Moreover, the gas separation performance of Br-BPDA-derived PIM-PIs improved with the ageing time, indicative their excellent resistance to physical ageing due to the relatively high chain rigidity resulting from the presence of *ortho*-substituted -Br and -CH_3_ groups. This work provides a new insight into advancing the design of PIM-PIs with long-term stability for gas separations.

## Figures and Tables

**Table 1:** Physicochemical properties of Br-BPDA-derived PIM-PIs.

Polymer	*T*_g_ (°C)	*T*_d5_ (°C)	*M*_w_ (kg mol^−1^)	PDI	Tensile Strength (MPa)	Modulus (GPa)	Elongation at Break (%)
Br-BPDA-MMBMA	416	443	81.0	1.9	59.2 ± 7.0	2.1 ± 0.1	3.4 ± 0.6
Br-BPDA-MMBDA	432	454	42.0	1.9	63.1 ± 0.5	2.0 ± 0.1	3.7 ± 0.2
Br-BPDA-TBDA1	419	416	45.0	1.9	78.5 ± 3.6	2.2 ± 0.1	4.9 ± 0.1
Br-BPDA-TBDA2	428	417	31.0	1.8	75.8 ± 5.6	1.8 ± 0.1	7.4 ± 1.4
Br-BPDA-DAT	>500	538	52.0	3.8	109.3 ± 7.0	2.1 ± 0.1	12.3 ± 1.1

**Table 2:** The microstructure of Br-BPDA-derived PIM-PIs.

Polymer	*S*_BET_ (m^2^ g^−1^)	CO_2_ Uptake (cm^3^ g^−1^)	*D* ^1^ (nm)	V_M_^2^ (cm^3^ g^−1^)	*d*_A_ (Å)	*d*_B_ (Å)	V_w_^3^ (cm^3^ mol^−1^)	*ρ* (g cm^−3^)	*FFV*
Br-BPDA-MMBMA	255	26.1	0.56/0.75/0.82	0.030	5.51	3.76	456.57	1.328	0.190
Br-BPDA-MMBDA	342	28.1	0.58/0.78/0.86	0.037	5.88	3.94	470.17	1.283	0.216
Br-BPDA-TBDA1	211	29.3	0.54/0.78/0.86	0.043	4.92	3.75	304.35	1.463	0.169
Br-BPDA-TBDA2	239	30.4	0.58/0.77/0.86	0.045	5.69	3.92	304.35	1.448	0.177
Br-BPDA-DAT	232	26.4	0.58/0.79/0.86	0.039	5.23	3.76	301.42	1.449	0.189

^1^ Pore width. ^2^ Total volumes of micropores. ^3^ Van der Waals volume, calculated using the Bondi’s group contribution method [35].

**Table 3:** Gas separation performances of Br-BPDA-derived PIM-PIs.

	Permeability (Barrer ^1^)					Selectivity				
Polymer	H_2_	CO_2_	O_2_	N_2_	CH_4_	H_2_/CH_4_	H_2_/N_2_	O_2_/N_2_	CO_2_/N_2_	CO_2_/CH_4_
Br-BPDA-MMBMA	270.0	315.5	55.1	14.1	19.0	14.2	19.1	3.90	22.3	16.6
300 days aged	198.0	190.7	32.2	7.3	9.1	21.8	27.1	4.4	26.1	21.0
900 days aged	147.9	136.3	21.7	4.4	4.7	31.5	33.6	4.9	31.0	29.0
Br-BPDA-MMBDA	576.5	724.5	143.2	42.9	61.7	9.4	13.4	3.3	16.9	11.8
300 days aged	380.6	476.9	81.3	20.1	27.3	13.9	18.9	4.0	23.7	17.5
900 days aged	321.9	399.0	62.3	15.1	19.0	16.9	21.3	4.1	26.4	21.0
Br-BPDA-TBDA1	200.4	161.6	27.7	6.2	7.3	27.6	32.5	4.5	26.2	22.2
300 days aged	105.3	63.2	11.8	2.3	2.3	45.8	45.8	5.1	27.5	27.5
900 days aged	73.2	36.9	6.7	1.2	1.0	73.2	61.0	5.6	30.8	36.9
Br-BPDA-TBDA2	561.6	711.0	127.0	40.7	51.3	11.0	13.8	3.1	17.5	13.9
300 days aged	370.9	449.1	78.6	19.3	26.3	14.1	19.2	4.1	23.3	17.1
900 days aged	240.5	284.1	45.6	11.0	12.9	18.6	21.9	4.1	25.8	22.0
Br-BPDA-DAT	349.8	384.4	69.8	16.3	19.7	17.7	21.4	4.3	23.5	19.5
300 days aged	204.3	170.7	28.8	5.9	6.6	31.0	34.6	4.9	28.9	25.9
900 days aged	109.8	89.0	15.3	2.8	2.7	40.7	39.2	5.5	31.8	33.0

^1^ 1 Barrer = 10^−10^ [cm^3^ (STP) cm]/(cm^2^ s cm Hg).

## Data Availability

Data are contained within the article.

## Supplementary Materials

The following supporting information can be downloaded at: https://www.mdpi.com/article/10.3390/polym16091198/s1, Figure S1: FT-IR spectra of Br-BPDA-derived PIM-PIs; Figure S2: ^1^H NMR spectra of Br-BPDA-derived PIM-PIs; Figure S3: ^13^C NMR spectrum of Br-BPDA-DAT in DMSO-*d*_6_; Figure S4: Stress–strain curves of Br-BPDA-derived PIM-PIs; Figure S5: Data of water contact angle on the Br-BPDA-derived PIM-PIs surface. Table S1: Solubility of Br-BPDA-derived PIM-PIs; Table S2: List of abbreviations, acronyms, and symbols.
