# Supplementary material for: Intrinsically Microporous Polyimides Derived from 2,2′-Dibromo-4,4′,5,5′-bipohenyltetracarboxylic Dianhydride for Gas Separation Membranes
Source: Polymers (Basel). 2024 Apr 25;16(9):1198. doi: 10.3390/polym16091198 (PMC11085140; doi:10.3390/polym16091198)
Supplement: Supplementary file 1 [file polymers-16-01198-s001.zip › polymers-2952427-supplementary.pdf]

Intrinsically Microporous Polyimides Derived from 2,2′-Dibromo-4,4′,5,5′-bipohenyltetracarboxylic Dianhydride for Gas Separation Membranes

Yongle Li [1,2], Yao Lu [3], Chun Tian [1,*], Zhen Wang [1] and Jingling Yan [1]

[1] Ningbo Institute of Material Technology & Engineering, Chinese Academy of Sciences, Ningbo 315201, China; liyongle@nimte.ac.cn (Y.L.); wz@nimte.ac.cn (Z.W.); jyan@nimte.ac.cn (J.Y.)
[2] College of Chemical Engineering, Zhejiang University of Technology, Hangzhou 310014, China
[3] School of Fine Arts, Zhengzhou University, Zhengzhou 450001, China; luyao94@zzu.edu.cn
* Correspondence: tianchun@nimte.ac.cn

List of Contents for Supplementary Materials:

Figure S1. FT-IR spectra of Br-BPDA-derived PIM-PIs
Figure S2. ^{1}H NMR spectra of Br-BPDA-derived PIM-PIs
Figure S3. ^{13}C NMR spectrum of Br-BPDA-DAT in DMSO-d_6
Figure S4. Stress-strain curves of Br-BPDA-derived PIM-PIs
Figure S5. Data of water contact angle on the Br-BPDA-derived PIM-PIs surface
Table S1. Solubility of Br-BPDA-derived PIM-PIs
Table S2. List of abbreviations, acronyms and symbols

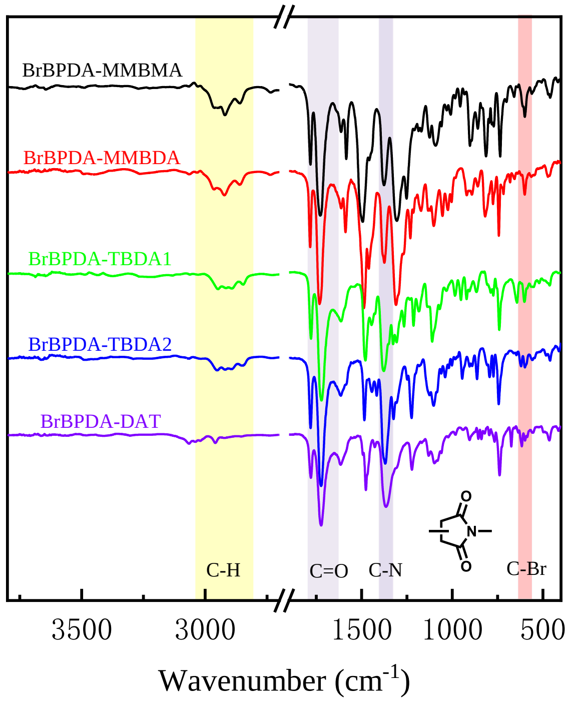

Figure S1. FT-IR spectra of Br-BPDA-derived PIM-Pis

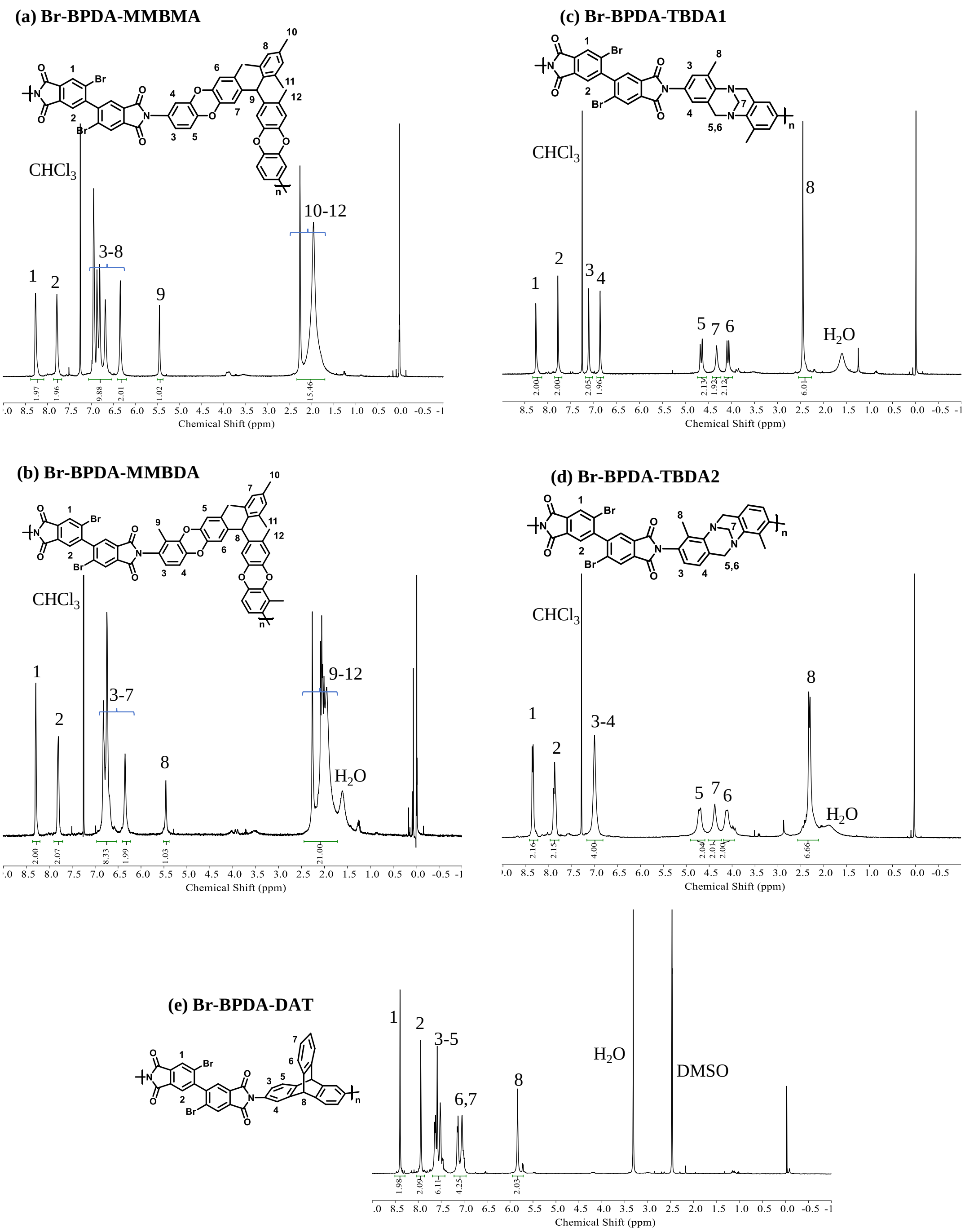

Figure S2. ^{1}H NMR spectra of Br-BPDA-derived PIM-PIs

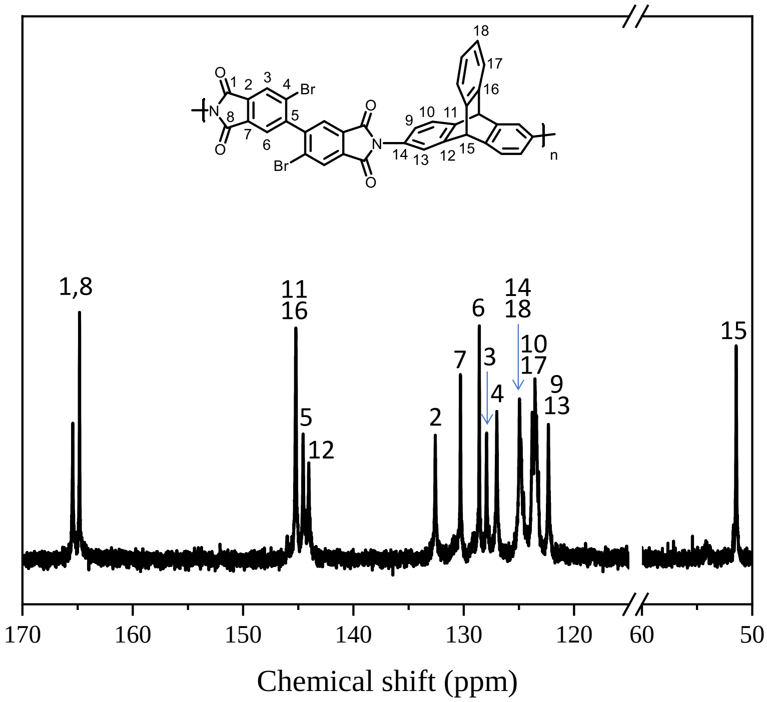

Figure S3. ^{13}C NMR spectrum of Br-BPDA-DAT in DMSO-d_6

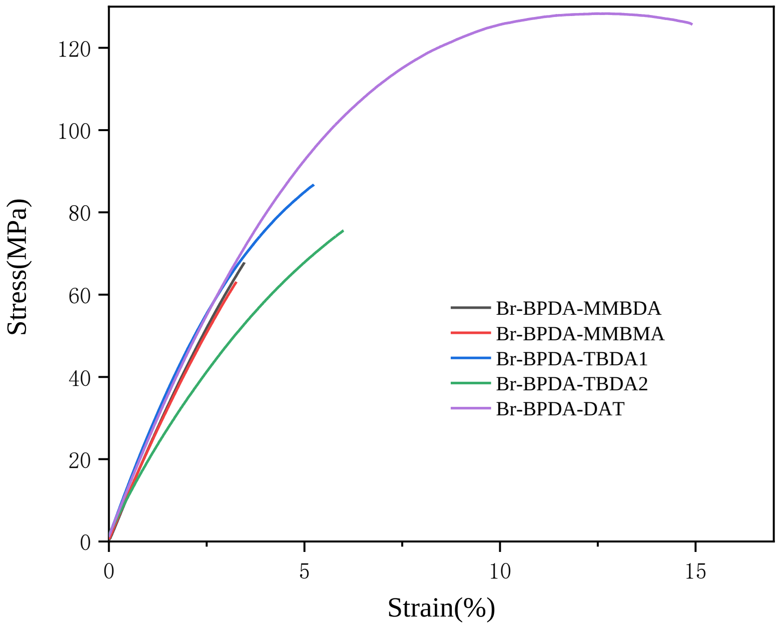

Figure S4. Stress-strain curves of Br-BPDA-derived PIM-Pis

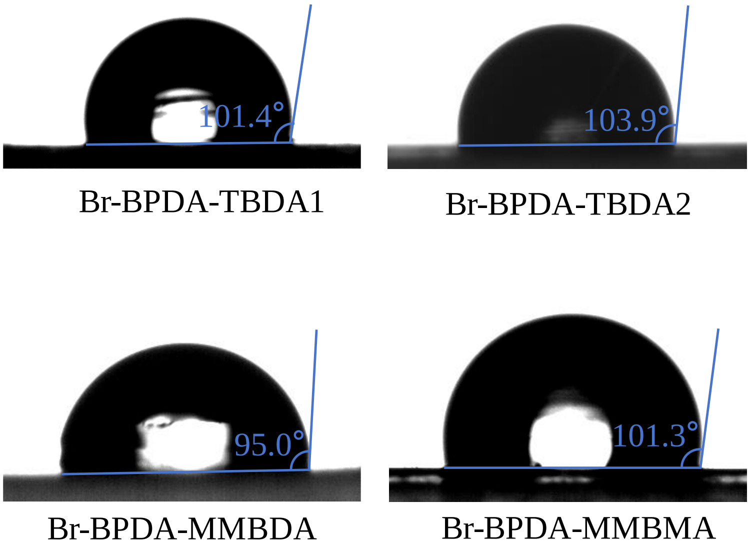

Figure S5. Data of water contact angle on the Br-BPDA-derived PIM-PIs surface

Table S1. Solubility of Br-BPDA-derived PIM-PI[a]

Polymer	*m*-Cresol	NMP	DMAc	DMF	DMSO	THF	CH_2Cl_2	$CHCl_3$
Br-BPDA-MMBMA	++	+-	++	++	+-	++	++	++
Br-BPDA-MMBDA	++	+-	++	++	+-	++	++	++
Br-BPDA-TBDA1	++	++	++	++	++	--	+-	++
Br-BPDA-TBDA2	++	++	++	++	++	--	+-	++
Br-BPDA-DAT	++	++	++	++	++	--	--	+-

[a] Determined at a concentration of 0.1g dL^{-1}. ++. Soluble at room temperature. +-, soluble upon heating. --, insoluble at room temperature.

Table S2. List of abbreviations, acronyms and symbols

Acronyms	Full name
4,4'-BPDA	3,3',4,4'-biphenyltetracarboxylic dianhydride
6FDA	4,4'-(Hexafluoroisopropylidene)diphthalic anhydride
Br-BPDA	2,2'-dibromo-4,4',5,5'-bipohenyltetracarboxylic dianhydride
D	Pore width
d_A	Peak A of *d*-spacing
DAT	(9R,10R)-9,10-dihydro-9,10-[1,2]benzenoanthracene-2,6-diamine
d_B	Peak B of *d*-spacing
DMA	Dynamic thermomechanical analysis
DMF	*N,N*-Dimethylformamide
FFV	Fractional free volume
FT-IR	Fourier transform infrared
GPC	Gel permeation chromatography
MMBDA	7,7'-(Mesitylmethylene)bis(1,8-dimethyldibenzo[b,e][1,4] dioxin-2-amine)
MMBMA	7,7'-(mesitylmethylene)bis(8-methyldibenzo[b,e][1,4]dioxin-2-amine
M_n	Number average molecular weights
M_w	Weight average molecular weights
NMP	*N*-Methylpyrrolidone
ODA	4,4'-oxydianiline
PDI	Polydispersity indices
PI	Polyimide
PIM	Polymers of intrinsic
PIM-PI	Intrinsically microporous polyimides
PTFE	Polytetrafluoroethylene
SBET	BET surface area
SBI	Spirobisindane
TB	Tröger’s base

TBDA1	4,10-dimethyl-6H,12H-5,11-methanodibenzo[b,f][1,5]diazocine-2,8-diamine
TBDA2	4,10-dimethyl-6H,12H-5,11-methanodibenzo[b,f][1,5]diazocine-3,9-diamine
T_{d5}	5% thermal decomposition temperature
T_g	Glass transition temperature
TGA	Thermogravimetric analysis
Trip	Triptycene
V_M	Cumulative volume of micropores
V_w	Van der Waals volume
WAXD	Wide-angle x-ray diffraction
